# Intraoperative Nice knots assistance for reduction in displaced comminuted clavicle fractures

**DOI:** 10.1186/s12891-021-04348-9

**Published:** 2021-05-22

**Authors:** Fangning Hu, Xi Liu, Fanxiao Liu, Honglei Jia, Xiaolong Lv, Fengrui Wang, Shihong Xu, Juanjuan Yang, Lingfei Hu, Bomin Wang, Yongliang Yang

**Affiliations:** 1 Department of Orthopaedics, Shandong Provincial Hospital affiliated to Shandong First Medical University, No. 324, Road Jing Wu Wei Qi, Jinan, 250021 Shandong China; 2 Department of Orthopaedics, Juye People’s Hospital, Heze, Shandong China; 3 Department of Radiotherapy, Shandong Cancer Hospital and Institute, Shandong First Medical University and Shandong Academy of Medical Sciences, Jinan, China; 4 Department of Emergency Surgery, Linyi People’s Hospital, Shandong First Medical University and Shandong Academy of Medical Sciences, Linyi, China

**Keywords:** Nice knot, Displaced clavicle fractures, Reduction, Fixation

## Abstract

**Purpose:**

The Nice knots have been widely used in orthopedic surgeries to fix torn soft tissue and fracture in recent years. The study aims to investigate the clinical efficacy and prognosis of intraoperative and postoperative Nice Knots-assisted reduction in the treatment of displaced comminuted clavicle fracture.

**Methods:**

From Jan 2014 to Dec 2019, 75 patients diagnosed with unilateral closed displaced comminuted clavicle fracture were treated with open reduction and internal fixation (ORIF) in this study. Nice knot group (the NK group) included 38 patients and the other 37 patients were in the traditional group (the TK group). The time of operation and the amount of bleeding during operation were recorded. Post-operative clinical outcomes and radiographic results were recorded and compared between these two groups. The Visual Analogue Scale (VAS), Neer score, Rating Scale of the American Shoulder and Elbow Surgeons, Constant-Murley score and complications such as infection, nonunion, implant loosening, fragment displacement and hardware pain were observed in the two groups.

**Results:**

In the comparison between the two groups, there was no significant difference in age, sex, the cause of displaced clavicle fracture, and other basic information between the two groups. The operation time, intraoperative fluoroscopy time, and intraoperative blood loss were significantly reduced in the NK group (*P* < 0.01). There were 2 cases of plate fracture in the TK group. The follow-up results showed that there was no significant difference in VAS, Neer score, ASES, and Constant-Murley scores between the two groups.

**Conclusion:**

The use of Nice knot, in comminuted and displaced clavicle fractures can reduce intraoperative blood loss, shorten operation time, facilitate intraoperative reduction, and achieve satisfactory postoperative clinical results. This study demonstrates that Nice knot is a simple, safe, practical and effective auxiliary reduction method.

## Introduction

Nice Knot, a novel suture fixation technique that combines a doubled suture with a sliding knot and has replaced metallic wires and cables for bone fixation [1]. The doubled-suture Nice knot can also be tied over a double-button and has been used for treatment of proximal humerus fractures, patella fractures, small- or medium-sized wounds, and rotator cuff tears [2–4]. Nice knots, especially with FiberWire, have excellent biomechanical properties [4–6]. In addition to the simplicity, firmness and stability of self-locking, the key point is that Nice Knot can bind the contralateral free bone which is not easy to be exposed. To our knowledge, although the Nice Knot has been widely used in the cases of orthopaedic trauma, there have been rare reports on the application of Nice knots in displaced comminuted clavicle fractures. In our clinical practice, we find that the use of the nice knot in middle clavicle fractures does provide good clinical results.

The clavicle is the only skeletal structure that connects the upper extremity to the trunk, which is of great importance to the stability of the shoulder joint. Clavicle fracture is a common type of fracture, accounting for approximately 2.6 to 5% of fractures in adults [7], which are usually divided into three parts according to their locations. Central clavicle fractures are the most common type of clavicle fracture, accounting for approximately 81% of clavicle fractures, and are more likely to be displaced than fractures of the inner and outer third [8].

Non-surgical treatment has traditionally been used, even if displacement occurs [9, 10]. In addition, Neer et al. [11] revealed that the nonhealing rate for surgical treatment of clavicle fractures was about 4%, compared with a nonoperative rate of about 0.1%. While the treatment of displaced clavicle fractures is still controversial, numerical researchers support surgical treatment. Some studies of surgical versus conservative treatment of clavicle fractures have shown significant advantages for the surgical group in terms of fracture healing and functional recovery [12–16]. Regarding to the conventional indications for surgery such as shoulder impaction, floating shoulder, open fractures, and fractures with neurovascular complications, surgery is recommended for younger active patients with bone shortening exceeding 1.5 cm [17]. Proper reduction is not only beneficial to the aesthetic healing of clavicle fracture, but also to the stable fixation of clavicle and the consistency of shoulder joint, and to the recovery of postoperative function of patients. However, intraoperative reduction is not as simple as it seems.

There are several reduction and fixation techniques for the treatment of displaced clavicle fractures, including compression screw repair and wire binding. However, smaller pieces of bone are not suitable for screw fixation; larger pieces of bone cannot resist shear forces such as rotation, and excessive periosteal dissection increase the risk of nonunion. Factors such as displacement and shortening may be important factors in fracture healing, and the search for factors such as osteonecrosis can help us to choose between conservative and surgical treatment [18, 19]. The method of steel wire binding increased the risk of complications. In fact, we have become clinically accustomed to using sutures to bind the free bone fragments in a displaced clavicle fracture.

Therefore, the purpose of this study was to investigate the intraoperative and postoperative clinical efficacy and outcomes of using Nice knot as an auxiliary reduction technique in displaced comminuted clavicle fractures. The working hypothesis is that sutures with the self-locking sliding Nice knots have the beneficial effect of convenience in the treatment of displaced comminuted clavicle fractures.

## Materials and methods

This retrospective study was approved by the ethics committee of our institution. Patients were selected according to the type of Robinson IIB clavicle fractures. The inclusion criteria were as follows: (1) closed clavicle fractures; (2) the type of Robinson IIB clavicle fractures (according to the Robinson classification of clavicle fracture [11]) were included. (3) the surgical technique was ORIF with plate.

The exclusion criteria were as follows: (1) multiple fractures with open fractures; (2) conservative treatment; (3) Adolescents with clavicle fractures; (4) chronic clavicle fractures; (5) the patients had severe medical comorbidities, such as diabetes and other complications.

We conducted a retrospective analysis of the data of patients with unilateral comminuted displaced clavicle fracture treated by open reduction and internal fixation in our hospital From Jan 2014 to Dec 2019, 75 patients diagnosed as unilateral comminuted displaced clavicle fracture were treated with open reduction and internal fixation with plates. Patients were assigned to two different groups depending on their auxiliary reduction method, 38 patients were treated with Nice knot-assisted open reduction and internal fixation (ORIF), and 37 patients were treated with traditional ORIF as control group.

All patients were examined by X-ray and CT, and diagnosed during operation, fluoroscopy during operation, X-ray examination after operation, and follow-up after operation.

### Operative technique

**Fig. 1:**
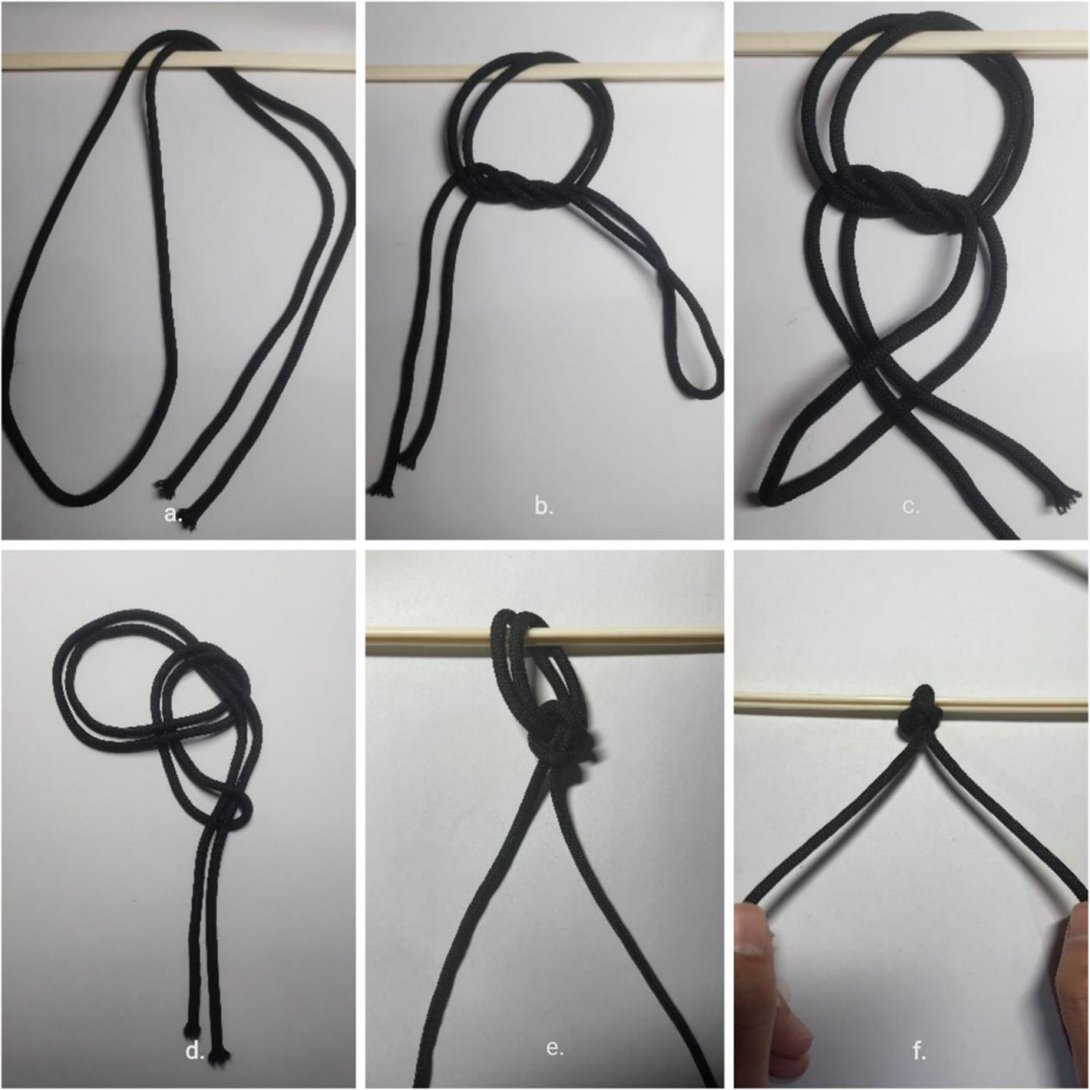
Nice Knot technique. **a** A doubled-over suture is passed around the tissue. **b** A single square knot is thrown. **c** The 2 free limbs are passed through the loop. **d** Illustration of Nice knot. **e** The knot is dressed. **f** The knot is slid down by pulling the 2 free limbs apart and the tightened knot is ready to be secured with 3 alternating half-hitches or surgeon’s knot

**Fig. 2:**
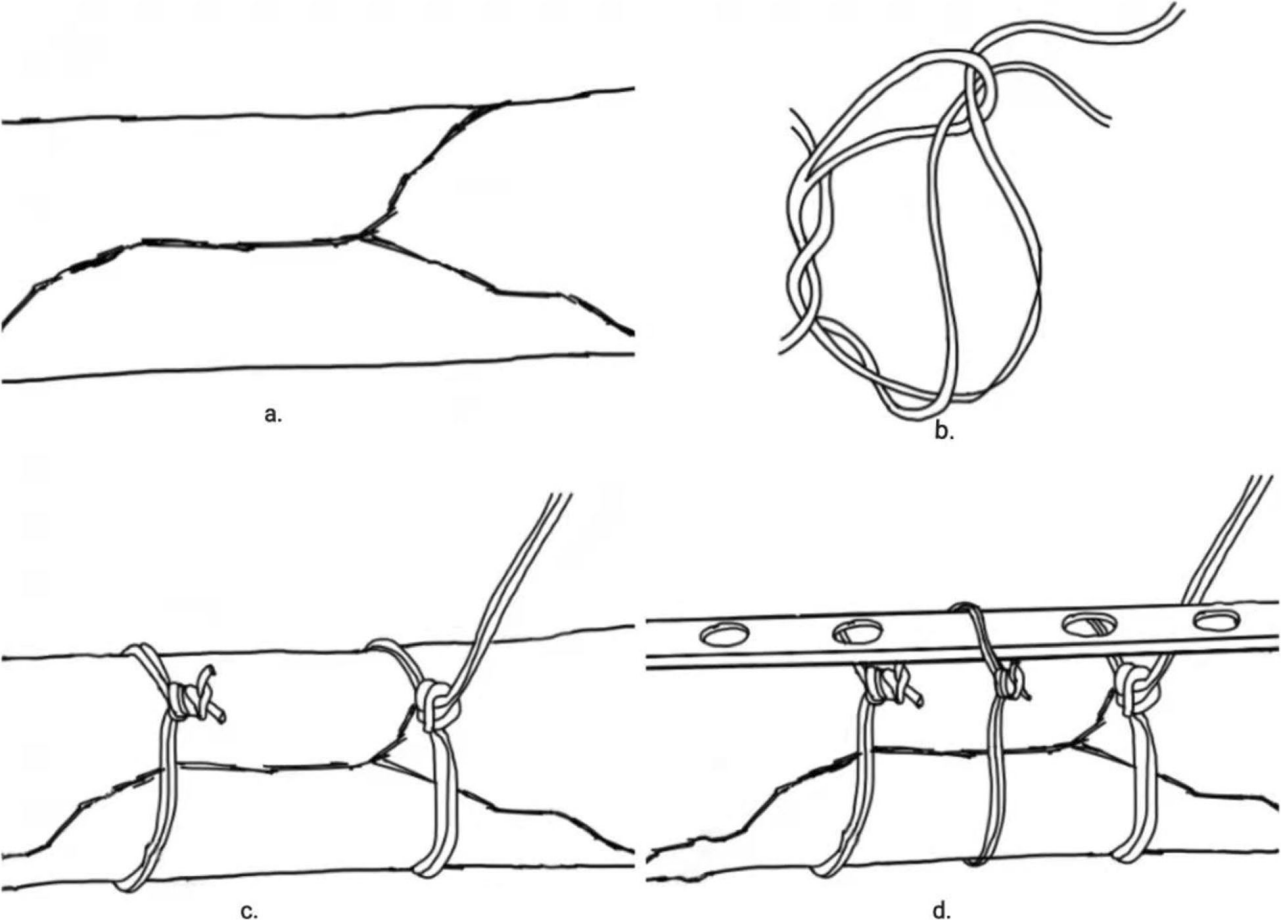
Illustration of the application of the Nice knots as an auxiliary fixation technique. **a** Displaced comminuted clavicle fracture. **b** Illustration of Nice knot. **c** Stable temporary reduction construct of fractured bone fragments using Nice knots. **d** After placing the plate, the Nice knot was followed to stabilize the fracture and the plate

**Fig. 3:**
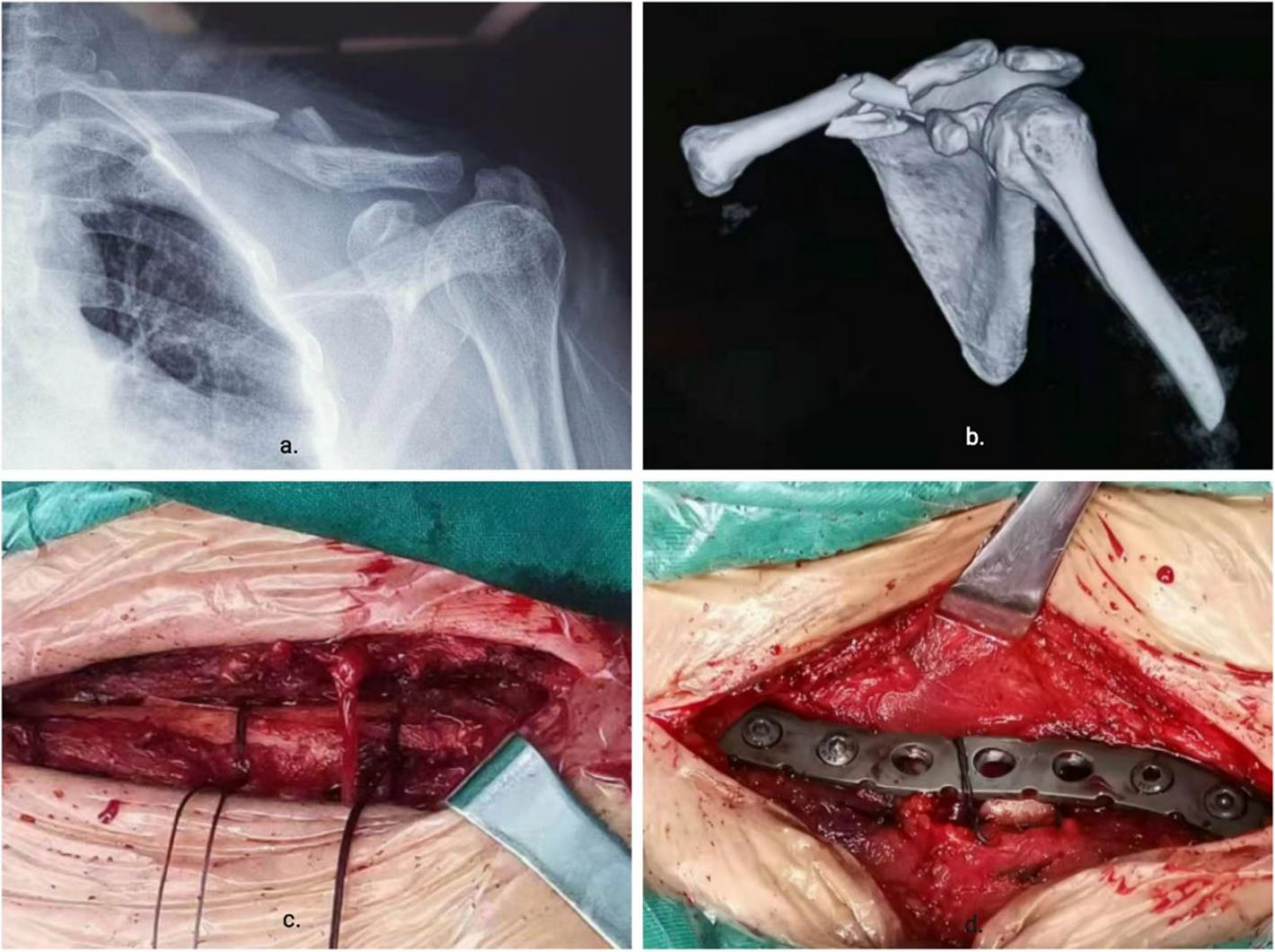
Intra-operative application of the Nice knots as an auxiliary reduction technique. **a, b** Pre-operative X-ray and CT indicate a displaced comminuted fracture of the clavicle. **c** Exposure of the main fracture line with the preservation of the rest of the periosteum and Nice knots were introduced surrounding the “Surrounding” of the fractured fragments and progressively tightened. **d** After proper placement of steel plates and screws, nice knot is used to strengthen the steel plates and clavicles

All cases were treated with general anesthesia by endotracheal intubation in supine position, and the operation was performed by the same surgical team. After the anesthesia was satisfied, the transverse incision parallel to the clavicle was used to open the skin and subcutaneous tissue and expose the fracture end with the broken end of the fracture as the center. In both groups, the fracture fragments or butterfly bones were temporarily fixed with reduction forceps. After a temporary reduction in the NK group, single needle and double-strand absorbable sutures were passed around the broken ends of the fracture. The suture was doubled over itself to obtain two free limbs on one end and a loop on the other. A simple square knot was thrown using the loop on one hand and two free limbs on the other. The loop was opened, and both free limbs were passed through it. The knot was then dressed by marking the smaller loop. The sliding knot was tightened by pulling the two free limbs apart. We have presented a series of photos to show a step-by-step instruction about the Nice knot(Fig. 1). The number of Nice Knots were determined according to the type of fracture and the size of the bone mass. The comminuted bone mass retains the surrounding soft tissue and is sutured to the trunk to reduce the bone defect at the broken end of the fracture as much as possible. After temporary reduction in the TK group, the larger sphenoid bone was fixed with screws alone. After temporary reduction, the appropriate length of anatomical locking plate was selected and placed above the clavicle in both groups. First, both sides of the broken end of the fracture were fixed with cortical nails, and the remaining two holes on both sides of the plate were fixed with locking screws. After inserting three screws at each end, the C-arm fluoroscopy showed that the reduction of the fracture, the position of the plate and the length of the screw were satisfactory. Due to the tension of the ligament around the clavicle, the shear force and rotation force between the clavicle and the steel plate make the broken end of the fracture lack stability, especially when the fracture line is long and the number of screws is limited. Therefore, the NK group continued to use single needle to guide single and double strand absorbable sutures around the broken end of the plate and clavicle, and sutured with Nice knot (Figs. 2 and 3).

### Pre- and post-operative management

**Fig. 4:**
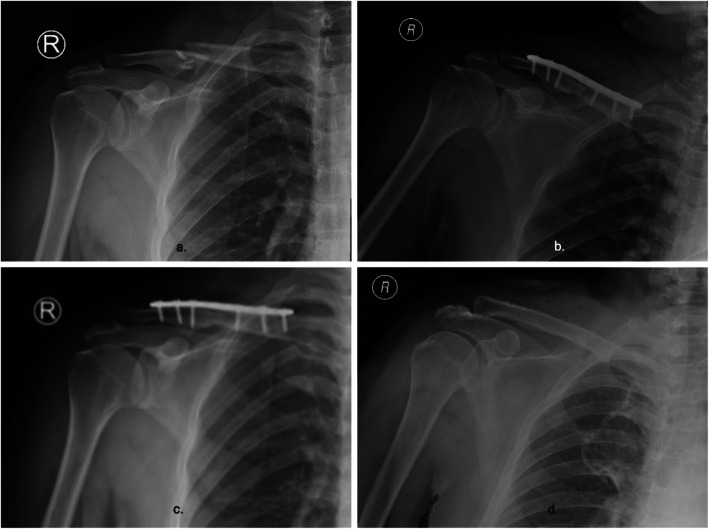
Imaging follow-up of Nice knot as an auxiliary reduction technique in the treatment of clavicle fracture. **a** The preoperative X-ray showed a displaced comminuted fracture of the clavicle. **b** On the first day after operation, routine X-ray examination showed that the alignment of the fracture was good. Three months after (**c**), the X-ray showed that the fracture healed well. Twelve months after (**d**), the internal fixation device was removed and X-ray examination showed that there was no fracture

Routine recording of the operation time (Min), from the beginning of skin incision to wound closure. The intraoperative blood loss (ml) was recorded. Positive X-ray films were taken on the 2nd day, 6th week, 3rd, 6th and 12th month after operation (Fig. 4). On the first day after operation, both groups began to fix the forearm sling for 3 weeks and carried out functional exercise, including passive functional exercise of the affected shoulder, active functional exercise of elbow joint, wrist joint and finger. All patients were able to move their shoulder joints freely during the follow-up of 6 weeks. At the last follow-up, shoulder function was evaluated according to visual analgesia score (VAS), Neer score, Rating Scale of the American Shoulder and Elbow Surgeons(ASES) and Constant-Murley score.

### Statistical analysis

We analysed the data using IBM SPSS Statistics 23.0 statistical software. Parametric data, such as operative time, fluoroscopic time, and blood loss, were described as mean ± standard deviation, and t-test was used for comparison between groups. Proportional values were compared using χ2-analysis or Fisher exact test where applicable. For each test, a *P*-value≤0.05 was considered significant.

## Results

There was no difference in age or sex between the two groups. All patients received surgical treatment within 48 h after admission and were discharged on the 3rd day after operation. The average follow-up of NK group was 13.1 ± 2.3 months (range 10 ~ 18 months) and TR group was 12.5 ± 2.6 months (range 08 ~ 18 months). Operative time was significantly shorter in the NK group (45.1 ± 3.8 [range 38–52] min), compared to the TK group (60.0 ± 7.3 [range 46–75] min, *p* < 0.01). The intraoperative blood loss in TK group was significantly higher than that in NK group (55.6 ± 7.7 ml vs. 84.0 ± 7.6 ml, *p* < 0.01). All patients in NK group healed smoothly (38/38,100%). In TK group, 35 cases healed (35/37, 95%). There were 2 cases of steel plate fracture in TK group (2/37 and 5.7%. (Tables 1 and 2).

**Table 1:** Patient demographics

Group	NK	TK	*P*
No. of cases	38	37	–
Gender (M/F)	21/17	19/18	0.734
Age (Year, mean ± SD)	35.3 ± 10.2	33.3 ± 10.0	0.3956
BMI	24.0 ± 2.4	24.7 ± 2.7	0.2304
History of tobacco and alcohol (Y/N)	12/26	10/27	0.665
Left or Right (L/R)	16/22	18/19	0.300
Injury mechanism			
Traffic accidents	18/20	15/22	0.551
Direct violent injury	12/26	14/23	0.569
Fall	8/30	8/29	0.952
Follow-up time	13.1 ± 2.3	12.5 ± 2.6	0.3164

**Table 2:** Operation related factors

Group	NK	TK	*P*
Operative time (min, mean ± SD)	45.1 ± 3.8	60.0 ± 7.3	< 0.01
Blood loss (ml, mean ± SD)	55.6 ± 7.7	84.0 ± 7.6	< 0.01
Fluoroscopy time (s, mean ± SD)	8.5 ± 1.3	14.0 ± 2.8	< 0.01
Hospitalization days(d, mean ± SD)	5.2 ± 1.9	6.3 ± 26	0.0469

There were no obvious postoperative complications in NK group, but there were 2 cases of plate fracture in TK group. In the comparison of postoperative functional score of fracture, Constant-Murley score at last follow-up were 96.7 ± 2.0 (range 93–100 points) in the NK group and 96.0 ± 2.1(range 92–100 points) in the TK group without a significant difference between groups. Neer score at last follow-up were 96.7 ± 1.9 (range 94–100 points) in the NK group and 96.0 ± 2.1(range 92–100 points) in the TK group without a significant difference between groups. Rating Scale of the American Shoulder and Elbow Surgeons at last follow-up were 96.2 ± 2.0(range 93–100 points) in the NK group and 96.0 ± 1.8 (range 93–100 points) in the TK group without a significant difference between groups. At the last follow-up, there was no significant difference between TR group (mean visual function score: 1.4 ± 1.2) and NK group (mean range of motion: 1.6 ± 1.0) (*P* > 0.05). (Table 3).

**Table 3:** Postoperative follow-up results

Group	NK	TK	*P*
Fracture healing time (month, mean ± SD)	3.6 ± 1.2	4.0 ± 1.6	0.2328
Time to sport (week, mean ± SD)	2.7 ± 0.6	2.8 ± 0.7	0.613
Time to work (month, mean ± SD)	6.3 ± 1.3	6.4 ± 1.3	0.712
Complication	0	2	–
ASES (mean ± SD)	96.2 ± 2.0	96.0 ± 1.8	0.5899
Neer score (mean ± SD)	96.7 ± .79	96.0 ± 2.1	0.1015
Constant-Murley score (mean ± SD)	96.7 ± 2.0	96.0 ± 2.1	0.1429
VAS(mean ± SD)	1.4 ± 1.2	1.6 ± 1.0	0.6416

## Discussion

Since the Nice knot was proposed, it has been widely studied and applied; Ian Peeters [20] suggested that a non-metallic cerclage technique mainly in the upper limb might become the golden standard, while in the lower limb both metallic and non-metallic cerclage techniques were complementary and dependent on indication. The biomechanical [21] comparison showed that high-performance sutures provided an alternative to steel wire for cerclage fixation, which had certain clinical application value. A recent biomechanical study [5] demonstrated that the Nice knot, especially using fiber wire, was biomechanically superior to the surgeon’s and Tennessee slider knots. P Collin [4] et al. made a mechanical analysis of Nice knot, which provided an option for sliding and locking knots, which reduced the risk of elongation during dynamic stress. Two half hitches were recommended to ensure adequate knot security. In 2016, Pascal Boileau [1] described a novel suture fixation technique that combined a doubled suture with a sliding knot and had replaced metallic wires and cables for bone fixation. The doubled-suture Nice knot has been used for the treatment of proximal humeral fractures, rotator cuff injuries, and the small- or medium-sized wounds [9]. Especially recently, Mengcun Chen [2] systematically explained the effect of Nice Knots as an auxiliary reduction technique in displaced comminuted patella fractures. As far as we know, there are very few studies about the application of the Nice knots in the treatment of the displaced clavicle fractures [1].

Although the treatment of clavicle fractures without displacement or complex displacement remains controversial, in young patients with > 15 mm clavicle shortening in the frontal plane, and especially in case of comminution and displacement fracture, surgery should be considered, due to elevated risk of non-union [17]. In recent years, the treatment of clavicle fracture had made progress, especially in the surgical treatment of middle clavicle fracture. Many studies [9, 22, 23] suggested surgical treatment of comminuted displaced mid- clavicle fractures to reduce shoulder discomfort caused by nonunion and malunion. Carlo Biz [24] et al. suggest endovascular treatment of pseudoaneurysms followed by internal fixation of clavicle fractures for co-morbid superannuated patients. The technique needed to take account of clavicle anatomy: especially periosteal vascularization in midshaft fracture. This segmentation of vascularization was difficult to objectify in surgery and suggested that intraoperative midshaft periosteum loss should be minimized [17, 25]. For the surgical treatment of closed middle clavicle fracture, in order to reduce soft tissue trauma, people continue to study the surgical methods. Current surgical options included superior plating, anterior-inferior plating, dual plating, and intramedullary nail fixation. Since the clavicle fracture was fixed with Kirschner wire in 1950, the technique of locking intramedullary nail had been developing continuously, indirect reduction could be used to fix the fracture end with intramedullary nail, so as to reduce the damage of soft tissue and blood supply at the fracture end, which could achieve satisfactory clinical effect [26]. Some clinical studies [27, 28] had shown that major re-intervention and re-fracture after implant removal occurred more frequently after plate fixation of comminuted displaced midshaft clavicle fractures. There were no significant differences between plate fixation and intramedullary fixation in terms of function and nonunion. However, for comminuted unstable fractures, plate treatment had a greater advantage. In recent literature, [9] the operative treatment of clavicle fractures showed lower rates of long-term sequelae, specifically lowering the incidence of symptomatic malunion and nonunion and improving functional outcomes. Some literatures [17] believe that plate osteosynthesis is the gold standard treatment for displaced midshaft fractures (DMCF). In the segmental defect model of clavicle fracture [29], the locking plate increases the torsional stiffness compared with the standard plate. This study described a simple, practical, and effective method to assist in the incisional repositioning of mid-clavicle comminuted displaced fractures based on the principles of reducing periosteal dissection and blood transport disruption and achieving anatomical repositioning as much as possible. In this study, the clinical results and functional outcomes of the displaced clavicle fractures with ORIF were good in both groups at the last follow-up.

The advantage of Nice knot is that it does not destroy the blood flow around the fracture like steel wire, and it can give satisfactory reduction to the subclavian fracture fragments. As we predicted, the Nice knot assisted reduction group could achieve better anatomical reduction without excessively peeling off the periosteum, and track the operation time, intraoperative blood loss, and intraoperative fluoroscopy time. We found that the operation time and intraoperative fluoroscopy time in NK group were significantly shorter than those in TK group (*P*<0.01), and not only that, we also found that the intraoperative blood loss in NK group was also significantly less than that in TK group (*P*<0.01). Therefore, Nice knots is promising method for the fixation of displaced fracture of clavicle fracture.

As we predicted, in the comparison between the two groups, the Nice knot assisted reduction group can achieve better anatomical reduction without excessive stripping of the periosteum, while reducing intraoperative blood loss and shortening the operation time. The Nice knots were used to stabilize the end of the fracture according to the type of fracture. For simple displaced fracture, the instrument was used to stabilize the fracture end and then bind 1–2 Nice knots around the fracture end. Nice knot had its special significance for comminuted and displaced fractures. Martijn hulsmans [30] reviewed the biomechanical studies of clavicle fractures, then pointed out that the loss of cortical line in wedge-shaped fracture and comminuted fractures directly affected the stability of intramedullary and plate fixation. On the other hand, the smaller fracture block screws were not suitable, the broken end nails were more likely to be loosened, and there was a greater risk of destroying the blood supply and affecting the healing of steel wire binding. The Saeed Asadollahi [31] study points out that some complications can be avoided by improving surgical techniques and careful use of cannulated screws for adequate fixation to obtain sufficient medial fragments. However, broken lines or butterfly-shaped bone blocks without treatment could result in bone defects and affect fracture healing. Blindly pursuing anatomical reduction and reduction was convenient. Excessive stripping of soft tissue around the fracture fragments would lead to the cutting off of blood supply of bone block, resulting in ischemic necrosis, delayed healing, nonunion and plate fracture. Recently, in order to reduce the damage of blood supply around the fracture, some studies [32, 33] have even shown that MIPPO technology can achieve good clinical effect by inserting clavicular plate, but it also loses the advantage of reducing complex fracture ends. The Kirschner wire or reduction forceps was firstly used to temporarily fix the main part of the fracture, and then bind it with Nice knots; finally, the remaining bone block should be kept as far as possible to keep its surrounding blood supply on the defect trunk. During our follow-up, we found two cases of plate fractures. Although there is no evidence to show the specific cause of clavicular plate fracture, we speculate that clavicular plate fracture may be related to coracoclavicular ligament traction. Anatomically, the injury of coracoclavicular ligament or acromioclavicular ligament can lead to shoulder instability. However, the stress at the broken end of the clavicle fracture is more concentrated because of the traction of these soft tissues in the middle clavicle fracture. Therefore, we added a Nice knot after placing the steel plate to stabilize the shear force between the fracture end and the steel plate. Of course, the corresponding evidence requires further biomechanical analysis.

Unlike the repair of proximal humerus, patella and rotator cuff injuries, clavicle fractures are mostly sutured and reduced under indirect vision. Therefore, we need to wind the clavicle during the operation, although we have not found any cases of subclavian vascular and nerve injury in our study, and many studies have pointed out that there are few intraoperative complications in the surgical treatment of clavicular fracture [31, 34]. However, during the operation, we still have to be very careful and pay attention to the course of the subclavian vessels and nerves in order to avoid damage during winding and drilling. In addition, the number of Nice knot used in clavicle fractures should be used reasonably to prevent excessive nodules from affecting the blood supply of the periosteum.

Although our data show that there are differences in operation time, intraoperative fluoroscopy time, intraoperative blood loss and fracture reduction satisfaction between the two groups, there are still some shortcomings in this study: 1. this study was a single-center retrospective clinical case analysis with a low level of evidence and a small number of cases, so multicenter, large sample case analysis will be needed to confirm the results of this study. It also illustrated the practicability and feasibility of Nice knot as a useful reduction tool in the treatment of displaced clavicle fracture. 2.This study was not randomly grouped, there was a certain selection bias. 3. Although the clinical and imaging effects of Nice knot were better than those of conventional reduction group, it was not clear whether this difference had clinical significance. And whether there are long-term complications, more long-term follow-up results are needed.

## Conclusion

The use of Nice knot, in comminuted and displaced clavicle fractures can reduce intraoperative blood loss, shorten operation time, facilitate intraoperative reduction, and achieve satisfactory postoperative clinical results. This study demonstrates that Nice knot is a simple, safe, practical and effective auxiliary reduction method.

## Data Availability

All data generated or analysed during this study are included in this published article.
